# Glutathione S-transferase A1 suppresses tumor progression and indicates better prognosis of human primary hepatocellular carcinoma

**DOI:** 10.7150/jca.36495

**Published:** 2020-01-01

**Authors:** Xiaojia Liu, Xianxian Sui, Canjing Zhang, Kelu Wei, Yun Bao, Ji Xiong, Zhongwen Zhou, Zhongqing Chen, Chaoqun Wang, Hongguang Zhu, Feng Tang

**Affiliations:** 1 Division of Surgical Pathology, Huashan Hospital, Fudan University, Shanghai 200040, China; 2 Laboratory of Medical Molecular Biology, Experimental Teaching Center, School of Basic Medical Sciences, Fudan University, Shanghai 200032, China; 3 Key Laboratory of Medical Molecular Virology, the Institutes of Biomedical Sciences, Fudan University, Shanghai 200032, China; 4 Shanghai Medical College, Fudan University, Shanghai 200032, China; 5 Department of General Surgery, Huashan Hospital, Fudan University, Shanghai, 200040, China; 6 Department of Pathology, School of Basic Medical Sciences, Fudan University, Shanghai 200032, China

**Keywords:** hepatocellular carcinomas, glutathione S-transferase A1 protein, prognosis, cellular proliferation, metastasis, AMP-Activated Protein Kinases

## Abstract

Glutathione S-transferase (GST) family members play an important role in detoxification, metabolism and carcinogenesis. The aim of this study is to investigate the effect of Glutathione S-transferase A1 (GSTA1) on the prognosis of HCC and to understand its role in tumor progression and the possible mechanism. GSTA1 in HCC was assessed using immunohistochemical staining, and it was found that HCC patients with better pathological differentiation had higher GSTA1 abundance. Further, high GSTA1 expression was correlated with low AFP, absent PVTT, and early stage TNM for HCC patients. Higher GSTA1 indicated longer overall survival and disease-free survival, while lower GSTA1 indicated poorer prognosis. Subsequently, lentiviral vector carrying GSTA1 gene was successfully constructed and maintained high expression in 97H and SNU449 liver cancer cells. We found that high GSTA1 restrained liver cancer cell proliferation, migration and invasion *in vitro*. Western blot showed that LKB1 and p-AMPK were upregulated while p-mTOR, p-p70 S6 Kinase and MMP-9 were downregulated in high GSTA1 groups. Taken together, high GSTA1 correlated with satisfactory prognosis of HCC. Additionally, GSTA1 may act as a protective factor through suppression of tumorigenesis by targeting AMPK/mTOR in HCC.

## Introduction

Primary hepatic cancer is the third leading cause of cancer-related mortality in China^1^. The most frequently occurring hepatic cancer is hepatocellular carcinoma (HCC), which accounts for 90-95% of all primary liver cancers and causes more than 234,000 deaths each year. Glutathione S-transferases (GSTs) are isoenzymes that have overlapping substrate specificities and protect cells from cytotoxic and carcinogenic agents^2^. Eight isoforms of cytosolic-soluble GSTs have been recognized in humans, including α, κ, μ, π, σ, θ, ζ, and ω^3^, ^4^. Glutathione *S*-transferase α1 (GSTA1, Gene ID: 2938) has shown both stimulatory^5^-^8^ and inhibitory effects^9^-^12^ on tumorigenesis. The association between genetic polymorphism of GSTA1 and susceptibility to cancer has been discussed in previous studies^13^-^15^, but unfortunately, the underlying mechanism has remained unclear. In this study, we aimed to clarify GSTA1's effect on HCC prognosis and to determine its role in tumor progression.

There were two phases of our GSTA1 investigation. In the first phase, we employed immunohistochemistry (IHC) to determine GSTA1 protein abundances in HCC tissues, and analyzed their correlations to HCC clinicopathological characteristics. We also studied the prognostic impact of GSTA1 with Kaplan-Meier survival curves and Cox regression analyses on HCC patients. In the second phase, we performed functional analysis by altering GSTA1 expression in liver cancer cells and performed cytology experiments to characterize its biological role in HCC progression and investigated the underlying mechanism.

## Materials and Methods

### Ethics statement

This investigation was conducted in accordance with the Declaration of Helsinki and guidelines approved by the Institutional Review Board of Huashan Hospital, with ratification from the National Natural Science Foundation of China (NSF No. 81902834).

### Patient selection

A total of 90 HCC patients who underwent hepatectomies during the year of 2011 were randomly selected from Huashan Hospital in our study. The criteria for case selection were as follows: (1) pathological diagnosis of HCC, (2) no anti-cancer therapies received prior to surgery, and (3) no history of other cancer. Tumor stage was defined according to the American Joint Committee on Cancer (AJCC, 2018-01-01, 8th edition) Tumor-Node-Metastasis (TNM) staging system. Tumor grade was assigned by the Edmondson-Steiner grading system.

All the patients were being regularly followed for up to 72 months, with a median survival time of 51 months (range, 1-72 months). The overall survival (OS) was defined as the length of time between the surgery and death, or the last follow-up examination. Disease-free survival (DFS) was calculated from the date of tumor resection until detection of tumor recurrence.

### TMA and Immunohistochemistry

Tissue microarray (TMA) was constructed and the slides were incubated with the rabbit polyclonal primary antibody against GSTA1 (NBP-33586, 1:2000 dilution, Novusbio, USA) overnight at 4°C in a moist chamber, and then conjugated with secondary antibody (NB7156, 1:2000 dilution; Novusbio, USA) for 60 minutes at room temperature. Finally, the slides were stained for 15 seconds using the DAB Kit (Boster Bio-Engineering Company, Wuhan, China). DAB staining regions for GSTA1 were scored by two pathologists blinded to the clinical parameters.

The score standard for the staining intensity was as follows: 0(negative), 1 (weak), 2 (moderate), 3 (strong). The score of staining extent was 1 (<25%), 2 (25%-50%), 3 (51%-75%), and 4 (>75%). The final GSTA1 expression score was calculated with the intensity score × extent score, ranging from 0 to 12^16^. The staining results were divided into 3 categories based on the sum of scores: 0-3 was low GSTA1 group, 4-8 was moderate GSTA1 group, 9-12 was high GSTA1 group.

### Cell culture and transfection

Human liver cancer cell lines (HepG2, 97H, SMMC-7721, HCC-LM3, PLC-PRF5, SK-Hep1 and SNU449) were obtained from Shanghai Institute of Cell Biology, Chinese Academy of Sciences. SNU449 was cultured in RPMI-1640 (Lot.1869036, Gibco, US), while others were cultured in Dulbecco's Modified Eagle Medium (DMEM, Cat No. 8113262, Gibco, US), supplemented with 10% fetal bovine serum (FBS, Lot.1438121, Gibco, US), penicillin (100 U/ml, Cat No.15140-122, Invitrogen, US), and streptomycin (100 mg/ml, Cat No.15140-122, Invitrogen, US), at 37°C in a 5% humidified CO_2_ incubator. The lentivirus vectors for upregulation of GSTA1 were obtained from GenePharma Co., Ltd (Shanghai, China). Liver cancer cells were infected with the lentivirus vectors constitutively expressing GSTA1 or empty vectors, according to the procedures of the manufacturer. Transfection efficacy was confirmed by western blot.

### Cell proliferation assay

The effect of GSTA1 on liver cancer cell proliferation was detected by CCK8. Cells in the logarithmic phase of growth were seeded in 96-well plates (1×10^3^/well) and cultured for 24, 48, 72 and 96 hours. Subsequently, 10 μL of CCK-8 solution (Dojindo Laboratories, Kumamoto, Japan) were added into each well and incubated for 2 hours. Optical density (OD) was measured at a wavelength of 450 nm by an automatic microplate reader (Bio Tek, USA). As for colony formation assay, cells were placed in six-well plates at 2×10^3^/well in triplicate and were routinely cultured for 2 weeks. Colonies were fixed by paraformaldehyde, stained in crystal violet, then photographed. The colonies were lysed with glacial acetic acid solution (GAAS, Cat No. 537020, Sigma-Aldrich, US), and the lysate concentration was quantified at an absorbance of OD560 nm using an automatic microplate reader^17^.

### Migration and Invasion assay

Transwell chambers were used to evaluate migration and invasion ability. For the migration assay, 5×10^4^ tumor cells were seeded in the upper chamber with serum free medium, while the lower chamber contained 10% FBS medium, and incubated for 24 h. For the invasion assay, the inserts were pre-coated with extracellular matrigel (2 μg/μL, BD USA) at 37°C for one hour and then 5×10^4^ cells were seeded into each well. 24 hours later, the media was discarded and the upper chamber was washed with PBS three times. The membrane was fixed in 4% polycondensation formaldehyde solution for 10 min and then stained with 0.5% crystal violet for 20 min, washed with water and dried 24 h before imaging. A 400-fold inversion microscope was used to count cells that moved to the sublayer of polycarbonate membranes.

### Western Blot Assay

Protein samples were extracted and then separated with SDS-PAGE and transferred onto nitrocellulose membranes (Bio-Rad, Hercules, USA). The membranes were blocked with 5% non-fat milk in Tris-buffered saline (TBS) containing 0.1% Tween-20 for 2 hours at room temperature. The blots were probed with the relevant primary antibodies overnight at 4°C, and then probed with a secondary antibody for 1 hour. An enhanced chemiluminescence detection method (Pierce ECL Western Blotting Substrate, Thermol, USA) was used to visualize the blots. Anti-GSTA1 (NBP-33586, 1:1000 dilution) was purchased from Novusbio company, USA. Other primary antibodies were purchased from CST company, including anti-AMPK α (#2795), anti-p-AMPK α Thr172 (#50081), anti-p-mTOR Ser2448 (#2971), anti-p-p70 S6 Kinase (#9204), anti-MMP-9 (#13667), and anti-LKB1 (#3050). Anti-β-actin (#3700) was used as the internal control antibody.

### Statistical Analysis

All experiments were repeated at least three times. Data were analyzed with SPSS software and expressed as mean ± SD. *P* < 0.05 was considered statistically significant. GSTA1 abundances between tumor and para-tumor tissue were analyzed by Wilcoxon signed-rank test. Kruskal-Wallis one-way analysis of variance (ANOVA) was performed to determine the relevance between GSTA1 and clinicopathological variables of HCC patients. Kaplan-Meier and log-rank test analyses were performed to determine the effect of GSTA1 on HCC patient survival. Multivariate Cox proportional hazard regression model was used to assess the prognostic variables in HCC. Mann-Whitney U test was performed to compare the variables of two groups in CCK8 assay, colony formation assay, invasion and migration assay, and western blot.

## Results

### High GSTA1 correlated with well-differentiation and early stage of HCC

**Figure 1:**
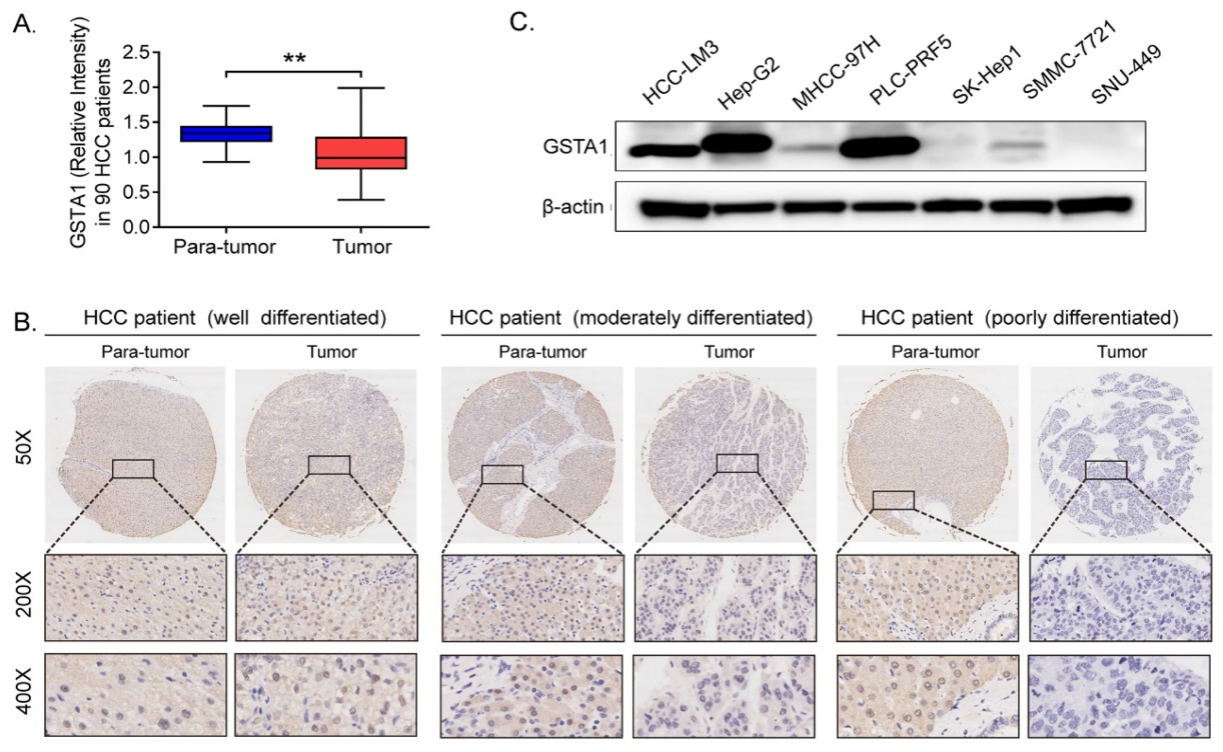
** GSTA1 decreased in HCC. A.** GSTA1 is downregulated in HCC tumor tissues compared with para-tumor tissues, calculated by Image Pro Plus (IPP) Image Analysis Software (**P < 0.01). **B.** the protein level of GSTA1 is related to pathological differentiation of HCC. GSTA1 was highly expressed in well-differentiated HCC tumors, but decreased heavily and was almost absent in poorly differentiated tumor tissues. Representative photomicrographs showed immunostaining of GSTA1 in well (*Left*), moderately (*Middle*) and poorly (*Right*) differentiated HCC specimens (magnification, 50×, 200×, 400×). **C.** Deficiency of GSTA1 was detected in liver cancer cell lines, including HCC-LM3 MHCC-97H, SK-Hep1, SMMC-7721 and SNU-449, but not in HepG2 and PLC-PRF5, checked by western blot.

IHC results indicated that GSTA1 was high in para-tumor tissues compared with that in HCC tissues (*P* < 0.05, Figure 1A). We also found that GSTA1 was related to the differentiation degree of HCC. The better the differentiation, the higher the expression of GSTA1, and vice versa (Figure 1B). And it was also very interesting that liver cancer cells with lower malignancy and weaker metastasis ability (including HepG2 and PLC-PRF5) had higher GSTA1 compared with other cells, which showed higher malignancy and strong metastasis ability (Figure 1C).

Further, our clinicopathologic characteristics study showed that high GSTA1 was correlated with low serum AFP, absence of PVTT, and early stage of TNM (Table 1, all *P* < 0.05). However, GSTA1 was not related to HCC patients' age, gender, HBsAg, tumor number or tumor size.

### Higher GSTA1 indicated better OS and DFS

**Figure 2:**
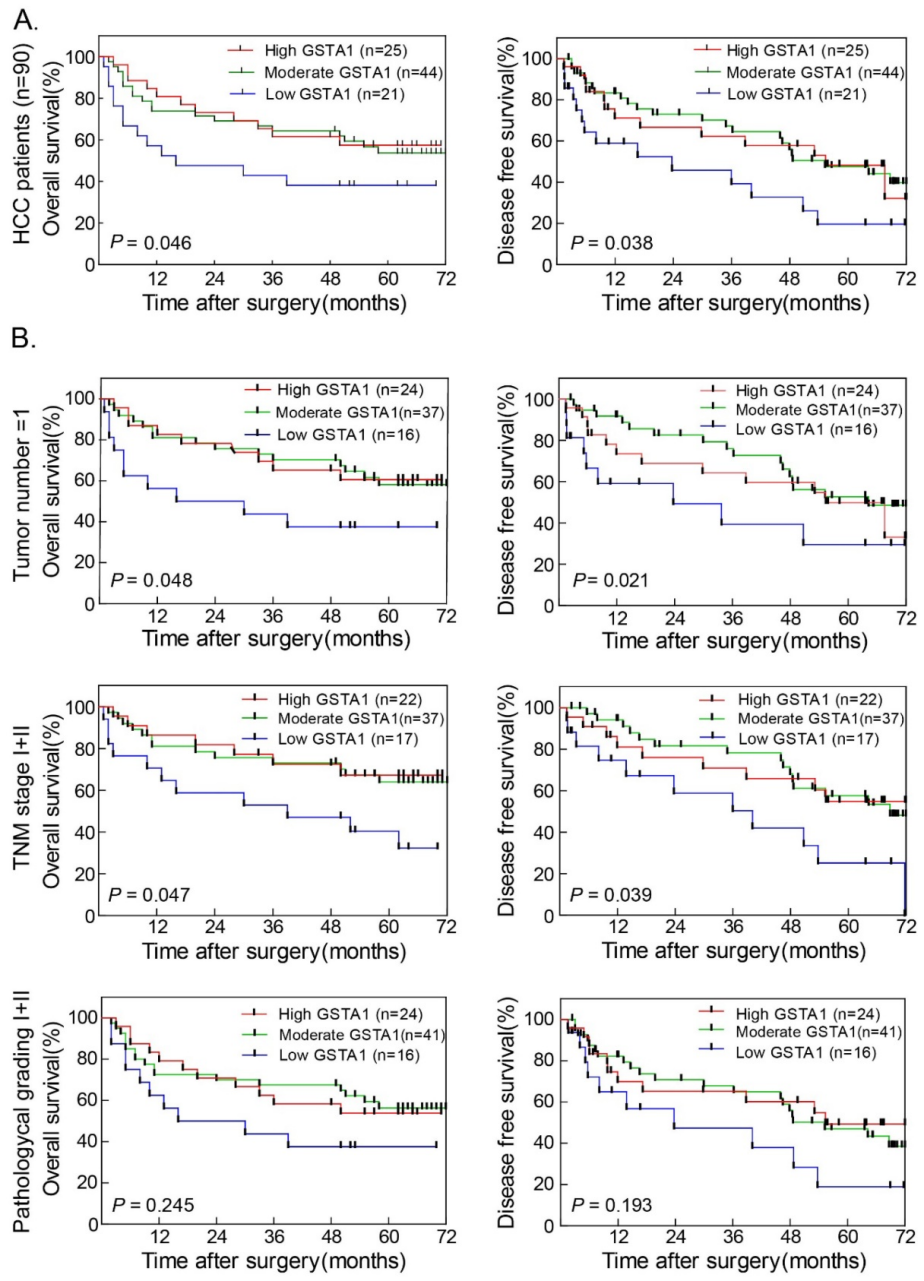
** Higher GSTA1 indicated better OS and DFS in HCC. A.** Patients with higher GSTA1 had longer OS and DFS, while lower GSTA1 indicated shorter OS and DFS (*P < 0.05). **B.** In single tumor number subgroup and TNM early stage subgroup, patients with high GSTA1 had long OS and DFS time (all *P < 0.05). The pathological grade I+II subgroup showed the same trend, but without statistical significance (*P* = 0.245 for OS and *P* = 0.193 for DFS, respectively).

In 90 HCC cases with prognostic information, we observed that GSTA1 was positively associated with OS (Figure 2A *Left*). Patients with higher GSTA1 had longer OS time, while low GSTA1 groups showed shorter OS. GSTA1 was also positively associated with DFS (Figure 2A *Right*). Patients with higher GSTA1 had longer DFS (median DFS = 64.27 months for high GSTA1 group and 55.37 months for moderate GSTA1 group), while lower GSTA1 patients had shorter DFS (median DFS = 23.73 months;* P* < 0.05).

The prognostic value of GSTA1 was further confirmed by stratified OS and DFS analyses. Higher GSTA1 was correlated with longer OS (Figure 2B *Left*) and DFS (Figure 2B *Right*) in single tumor number and TNM stage I+II (all *P* < 0.05). The pathological grade I+II subgroup showed the same trend, but without statistical significance.

### GSTA1 maybe an independent prognostic factor for HCC

Univariate analysis showed that GSTA1, AFP, tumor number, tumor size, PVTT, and TNM stage were related to OS (Table 2) and DFS (Table 3) in HCC patients. Multivariate analysis was performed using the Cox Proportional hazards model and the analysis revealed that GSTA1, AFP, tumor number, PVTT and TNM were independent prognostic factors for HCC (all *P* < 0.05).

### GSTA1 overexpression inhibited hepatic cancer cell proliferation

**Figure 3:**
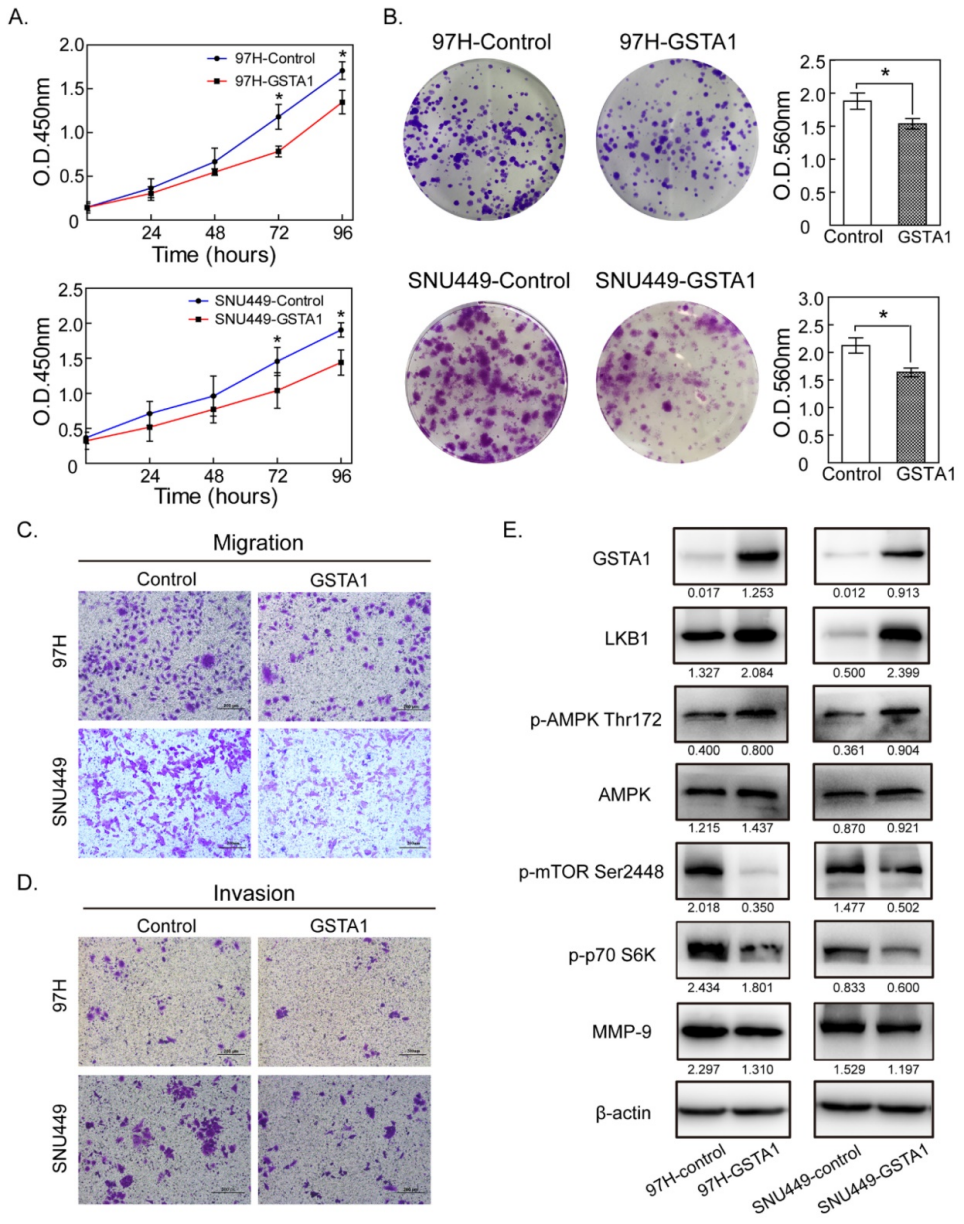
** GSTA1 overexpression decreased liver cancer cell proliferation, migration and invasion abilities. A.** CCK8 assay showed that GSTA1-transfected cells had decreased cell viability compared with empty vector control cells (*P < 0.05). **B.** Colony size and density in GSTA1 groups were smaller and rarer than those in control groups (*P < 0.05). The abilities of migration **(C.)** and invasion **(D.)** of liver cancer cells transfected with GSTA1 were decreased, which were detected by trans-well assays (*P < 0.05). Representative images were selected randomly from 5 fields (crystal violet staining, 400×) in each group and quantified by mean of five random fields. **E.** GSTA1 overexpression downregulated AMPK/mTOR. Western blot showed that GSTA1 protein was overexpressed in GSTA1-transfected liver cancer cells, indicating a successful transfection. In 97H and SNU449 cells, GSTA1 overexpression increased LKB1 and p-AMPK Thr172 protein expression while the total AMPK amount remained unchanged. Western blotting results indicated that p-mTOR 2448 decreased in cells overexpressing GSTA1, compared with controls. Besides, p-p70 S6K as well as MMP-9 were downregulated in the GSTA1 groups.

Liver cancer cells 97H and SNU449 (with a very low GSTA1) were chosen as experimental cells and were infected with the lentivirus vectors constitutively expressing GSTA1. CCK8 assays showed that OD450 of 97H in the control group was 0.363 ± 0.052, 0.666 ± 0.079, 1.179 ± 0.104 and 1.707 ± 0.127 at 24, 48, 72 and 96 hours, respectively. GSTA1 reduced the viability of 97H cells in a time-dependent manner, with OD450 of 0.303 ± 0.050, 0.547 ± 0.047, 0.784 ± 0.281, and 1.347 ± 0.072. A similar trend occurred in SNU449 cells (Figure 3A). Colony-formation assays were used to evaluate the long-term effect of GSTA1 on cell survival. GSTA1 overexpression led to a decrease in cell colony formation ability in 97H and SNU449 cells (all *P* < 0.05, Figure 3B).

### GSTA1 overexpression reduced hepatic cancer cell migration and invasion abilities

The migration and invasion abilities of liver cancer cells (97H and SNU449) were weakened in GSTA1 groups. Migration assays showed that the numbers of migratory cells were much less in GSTA1 groups than those in control groups. In invasion assays, the numbers of cells passed through the matrigel in GSTA1 groups were 26.00 ± 8.54 for 97H and 63.18 ± 4.22 for SNU449, and less than that in control groups (38.30±10.05 for 97H and 93.14±7.17 for SNU449), all *P* < 0.05, Figure 3C and 3D.

### GSTA1 may regulate the AMPK/mTOR pathway

In GSTA1-overexpression groups, western blot showed an increased abundance of GSTA1 compared with the control groups, indicating that GSTA1 had been expressed stably and effectively in 97H and SNU449. GSTA1 overexpression upregulated LKB1 and p-AMPKα Thr172, without too much effect on total AMPK, but downregulated p-mTOR Ser2448, p-p70 S6K and MMP-9 (all *P* < 0.05, Figure 3E).

## Discussion

Previous GSTA1 studies have reached contradicting conclusions, so we investigated GSTA1's effects on tumor progression and prognosis in HCC. We began our study by exploring GSTA1 in tissue microarray using IHC. Analysis showed that GSTA1 abundances were lower in HCC tissues than that in adjacent para-tumor liver tissues. And patients with poorer differentiation had much lower GSTA1. These results align with those of Hayes PC^18^ and Campbell JA^19^, which found decreased GSTA1 activity in HCC tissue. GSTA1 is a kind of toxicide that can expel cytotoxic or reactive oxygen species (ROS) from the body. At the beginning of tumorigenesis, ROS accumulate slightly, and could be eliminated by enzymatic or nonenzymatic antioxidant easily^20^, ^21^. However, with the progression of tumors, ROS increased, and antioxidants were suppressed in carcinoma^22^. Therefore, we speculated that the decrease of GSTA1 in advanced hepatocellular carcinoma may be mainly related to ROS accumulation which proved to be able to accelerate the progression of HCC^23^.

We also observed that high GSTA1 was correlated with PVTT absence and low serum AFP. Both these factors occur during the early stage of tumorigenesis. And the number of PVTT or the serum AFP increased concomitantly with tumor progression in most HCC cases^24^. Besides, we also found that GSTA1 was related to TNM stage in primary HCC. The earlier the stage, the higher the expression of GSTA1. The later the stage, the lower the expression of GSTA1. It was also very interesting that liver cancer cell lines with stronger metastasis ability had a lower GSTA1 abundance compared with other cells, which showed less metastatic potential. So, we suggested that GSTA1 may act as a biomarker in the progression of HCC, and the decrease of GSTA1 may indicate distant metastasis of the tumor and bad prognosis for HCC patients.

The clinical significance suggests that GSTA1 might influence the biological behavior of HCC. We found that GSTA1 suppresses hepatic cancer cell growth in both a short time and a long period, with a downregulation of p70 S6K (p70 ribosomal protein S6 kinases), which could promote elongation fator-1a (EF-1a) and Poly A-binding protein (PABP)^25^. Besides, compared with control groups, GSTA1 overexpression cells showed much weak mobility in migration and invasion assays, and decreased abundance of MMP-9. Matrix metalloproteinases (MMPs) can degrade almost all the components of extracellular matrix (ECM) and destroy the histological barrier during the invasion, and play an important role in the metastasis of tumors 26, 27. MMP-9 is upregulated in HCC and is a marker of a bad prognosis^28^. So, these results suggest that downregulation of MMP-9 caused decreased metastasis ability of GSTA1 overexpressed cells.

Liver kinase B1(LKB1), an important upstream gene of adenylate-activated protein kinase (AMPK), acts as a tumor suppressor^29^. Previous experiments have shown that LKB1 can activate AMPK and then negatively regulate mTOR, a key biological macromolecule that promotes cell metabolism and growth^30^. In the process of tumorigenesis, decrease or absence of LKB1 or p-AMPK could activate p-mTOR, which phosphorylates S6K and 4EBP1, accelerates cell cycle, enhances cell proliferation, and ultimately accelerates tumorigenesis.

Our results showed that GSTA1 overexpression decreased HCC cell proliferation and significantly reduced their migration and invasion. It has been reported that glutathione deficiency is associated with LKB1 loss under oxidative stress^29^. We speculated that as a vital assistant of GSH, GSTA1 may cause LKB1 upregulation, which activated AMPK and reduced p-mTOR, thereby inhibiting cell proliferation and migration and invasion. In this experiment, increased LKB1 had a significant effect on AMPK/mTOR/MMP-9. Zhuang, et al.^31^ also found that high expression of LKB1 could reduce the expression of MMPs and inhibit the metastasis of breast cancer cells. So, it is reasonable that LKB1 inhibits the metastasis of GSTA1 overexpressed liver cancer cells by downregulating the expression of MMP-9.

In summary, better pathological differentiation of HCC indicated higher GSTA1 in tumors. And patients with higher GSTA1 were more likely to have better prognosis or stay in early stage HCC. We found that GSTA1 overexpression could inhibit liver cancer cell proliferation and metastasis through regulating LKB1/AMPK/mTOR directly or indirectly. All these results indicated GSTA1 could be applied as a potential prognostic biomarker and a new therapeutic target in HCC.

## Figures and Tables

**Table 1:** Correlation between GSTA1 and clinicopathologic features in 90 HCC patients

Variable (missing cases)	Cases	GSTA1			*P*-value
		Low	Moderate	High	
Gender					0.524
Female	20(22.2%)	7(35.0%)	7(35.0%)	6(30.0%)	
Male	70(77.8%)	14(20.0%)	37(52.8%)	19(27.2%)	
Age/year					0.271
≤ 50	40(44.4%)	12(30.0%)	18(45.0%)	10(25.0%)	
>50	50(55.6%)	9(18.0%)	26(52.0%)	15(30.0%)	
HBsAg					0.928
Negative	19(21.1%)	8(42.1%)	9(47.4%)	2(10.5%)	
Positive	71(78.9%)	13(11.2%)	35(26.4%)	23(43.1%)	
Preoperative AFP					0.026*
≤ 400 ng/mL	56(62.2%)	7(12.5%)	32(57.1%)	17(30.4%)	
>400 ng/mL	34(37.8%)	14(41.2%)	12(35.3%)	8(23.5%)	
Tumor Number					0.055
Single	77(86.6%)	16(20.8%)	37(48.0%)	24(31.2%)	
Multiple	13(13.4%)	5(38.5%)	7(53.8%)	1(7.7%)	
Tumor size					0.307
≤ 3 cm	32(35.6%)	5(15.6%)	17(53.1%)	10(31.3%)	
3-5 cm	22(24.4%)	6(27.3%)	10(45.5%)	6(27.2%)	
> 5 cm	36(30.0%)	10(27.8%)	17(47.2%)	19(25.0%)	
PVTT					0.005**
Absent	58(64.4%)	9(15.5%)	31(53.4%)	18(31.1%)	
Present	32(35.6%)	12(37.5%)	13(40.6%)	7(21.9%)	
Differentiation					0.026*
Well	9(10.0%)	3(33.3%)	1(11.1%)	5(55.6%)	
Moderate	45(50.0%)	5(11.1%)	27(60.0%)	13(28.9%)	
Poor	36(40.0%)	13(36.1%)	16(44.4%)	7(19.5%)	
TNM Stage					0.011*
Ⅰ	56(62.2%)	6(10.7%)	32(57.1%)	18(32.2%)	
Ⅱ	20(22.2%)	11(55.0%)	5(25.0%)	4(20.0%)	
Ⅲ-Ⅳ	14(15.6%)	4(28.6%)	7(50.0%)	3(21.4%)	

*P<0. 05, **P<0.01. Abbreviations: GSTA1: Glutathione S-transferase A1. HBsAg: hepatitis B surface antigen. AFP: alpha-fetoprotein. PVTT: portal vein tumor thrombosis. TNM: tumor-node-metastasis.

**Table 2:** Univariate and multivariate analysis for predictors of OS in 90 HCC patients

Variables	OS			
	Univariate	Multivariate		
	*P*-value	HR	95%CI	*P*-value
Gender (Female* vs* Male)	0.234	-	-	-
Age/year (≤ 50 ys *vs* > 50 ys)	0.180	-	-	-
HBsAg (Negative *vs* Positive)	0.932	-	-	-
AFP (ng/mL) (≤ 400 *vs* > 400)	0.000**	2.641	1.387-5.025	0.003**
Number of tumors (Single* vs* Multiple)	0.022*	2.174	1.036-4.560	0.040*
Tumor size d/cm (≤ 5 *vs* > 5)	0.005**	1.635	1.137-2.351	0.008**
Pathological grade (Ⅰ vs Ⅱ *vs* Ⅲ-Ⅳ)	0.015*	1.752	1.044-2.942	0.034*
PVTT (Present *vs* Absent)	0.000**	2.805	1.503-5.236	0.001**
TNM (Ⅰ vs Ⅱ *vs* Ⅲ-Ⅳ)	0.000**	2.566	1.721-3.826	0.000**
GSTA1 (Low *vs* Moderate* vs* High)	0.036*	2.675	1.853-4.192	0.047*

*P<0.05, **P<0.01. Abbreviations: OS: overall survival. HR: hazard radio. CI: confidence interval. HBsAg: hepatitis B surface antigen. AFP: alpha-fetoprotein. PVTT: portal vein tumor thrombosis. TNM: tumor-node-metastasis. GSTA1: Glutathione S-transferase α1.

**Table 3:** Univariate and multivariate analysis for predictors of DFS in 90 HCC patients

Variables	DFS			
	Univariate	Multivariate		
	*P*-value	HR	95%CI	*P*-value
Gender (Female *vs* Male)	0.725	-	-	-
Age/year (≤ 50 ys *vs* > 50 ys)	0.150	-	-	-
HBsAg (Negative* vs* Positive)	0.847	-	-	-
AFP (ng/mL) (≤ 400 *vs* > 400)	0.016*	2.257	1.230-4.139	0.009**
Number of tumors (Single *vs* Multiple)	0.002**	3.361	1.621-6.969	0.001**
Tumor size d/cm (≤ 5 *vs* > 5)	0.034*	-	-	-
Pathological grade (Ⅰ *vs* Ⅱ *vs* Ⅲ-Ⅳ)	0.404	-	-	-
PVTT (Present *vs* Absent)	0.000**	3.971	2.182-7.227	0.000**
TNM (Ⅰ *vs* Ⅱ *vs* Ⅲ-Ⅳ)	0.000**	3.664	2.453-5.473	0.000**
GSTA1 (Low *vs* Moderate *vs* High)	0.040*	2.113	1.927-5.044	0.046*

*P<0.05, **P<0.01. Abbreviations: DFS: disease free survival. HR: hazard radio. CI: confidence interval. HBsAg: hepatitis B surface antigen. AFP: alpha-fetoprotein. PVTT: portal vein tumor thrombosis. TNM: tumor-node-metastasis. GSTA1: Glutathione S-transferase α1.
